# Perspectives on community-based system change for people living with persistent pain: insights from developing the “Rethinking Pain service”

**DOI:** 10.3389/fpain.2024.1299027

**Published:** 2024-03-20

**Authors:** Mark I. Johnson, Kerry Page, James Woodall, Kate Thompson

**Affiliations:** ^1^ Centre for Pain Research, School of Health, Leeds Beckett University, Leeds, United Kingdom; ^2^ Rethinking Pain Programme, Keighley Healthy Living (KHL) & Health Action Local Engagement (HALE), Bradford, United Kingdom; ^3^ Centre for Health Promotion, School of Health, Leeds Beckett University, Leeds, United Kingdom

**Keywords:** chronic pain, voluntary and community sector, system change, clinical sector, salutogenesis, whole health, community

## Abstract

In this perspective article we advocate community-based system change for people living with persistent pain. Our view is that greater use of the voluntary and community sector, in partnership with the clinical sector, creates the conditions for a “whole person” approach to pain management, leading to greater personalised care for adults living with long-term pain whilst having the potential to ease some of the pressures on General Practitioners and other clinical services. We advocate pain care that is socially connected, meaningful within socio-cultural contexts and aligned with the principles of salutogenesis. We provide an example of a UK National Health Service (NHS) commissioned pain service called “Rethinking Pain” that operationalises this perspective. Led by the voluntary and community sector, Rethinking Pain works in partnership with the clinical sector to provide a central holistic pathway of care for people experiencing persistent pain. This is the first time that this model of care has been commissioned for persistent pain in this area of England. The Rethinking Pain service is underpinned by core values to work with people to manage their pain holistically. The Rethinking Pain team proactively engage with people in the community, actively approaching and engaging those who experience the biggest health inequalities. In this article we provide an overview of the context of pain services in the UK, the rationale and supporting evidence for community-based system change, and the context, pathway, values, goals, and aspirations of the Rethinking Pain service.

## Introduction

Recently, the UK has introduced integrated care systems that focus on a person-centred approach as part of reforming its health and care landscape (1). Guided by the National Health Service (NHS) Long Term Plan, health and care services need to continually evolve to meet the changing health needs of society (2). A key area of work within the NHS long term plan is “personalised care” which focusses on shared decision making, giving people more choice and control over their mental and physical health. In 2023, the International Association for the Study of Pain (IASP) Global Year advocacy campaign focussed on integrative pain care (3), defined as “… the carefully planned integration of multiple evidence-based treatments—offered to an individual suffering from pain—that strives to be individualized (person-centred), mechanism-guided, and temporally coordinated”. This definition situates individualised person-centred care within a mechanistically guided treatment paradigm. Gaudet et al. contend that people need to be equipped to take control of their mental, emotional, spiritual, and physical health in order to live their most meaningful lives, and that this cannot be achieved solely by improvements in existing health systems (4). Instead, Gaudet et al. argue there is a need for “true cultural transformation” in health care services for whole health to be realised (4).

We advocate policies and practices that promote health at the community level (5, 6), utilising the principles of salutogenesis as a vehicle to alleviate painogenic environments and the burden of persistent (chronic) pain (7). Salutogenesis is about creating an environment and lifestyle that supports the way people understand their interaction in the world (comprehensibility), view their life (meaningfulness) and respond to stressful situations (manageability), i.e., developing a strong sense of coherence (8, 9). Salutogenic interventions take a whole-person approach grounded in a person's unique life story and current situation, encompassing physical, mental, social, and spiritual aspects that are meaningful to the individual. Through active participation, individuals learn to identify and utilise resources to successfully change comprehensibility, meaningfulness, and manageability, to reconfigure their conception of health as a lifelong process, with stressors and tension becoming a normal part of life and potentially health-promoting. In 2022, Langeland et al. reviewed 41 studies and concluded that salutogenic programs and interventions help people learn how to construct self-identity and a sense of coherence in various challenging life situations including mental health problems and pain (10). In other words, salutogenic approaches help people actively adapt to stressful environments through discovery of resources that shift them away from “dis-ease”.

In this article, we provide perspective for personalised care for people living with persistent pain through greater use of voluntary and community care sector activity. We describe a new community-based chronic pain support service called Rethinking Pain that is led and delivered by the voluntary and community sector. Rethinking Pain aligns with the key features of salutogenesis and whole-person health. Although a salutogenic framework was not used in the design of the service and a sense of coherence not measured as a health outcome, we contend that community-based support pain services, such as Rethinking Pain, are likely to improve sense of coherence not only in individuals, but also in communities. Rethinking Pain is commissioned by Bradford District and Craven Health and Care Partnership (a statutory NHS organisation responsible for the provision of health services in England), including funding from Primary Care Networks. We provide examples of the referral pathway along with the context, goals, values and aspirations of the Rethinking Pain service, from the perspective of the Rethinking Pain team, captured during one, two-hour focus group discussion, conducted six months after the service had “gone live”. The focus group discussion was conducted as part of a broader, on-going, service evaluation underpinned by Theory of Change research methodology (11) which we will publish in a subsequent research article. However, the Rethinking Pain team members provided informed consent that anonymised quotes could be used in this “Perspectives Article” as examples of their views.

## Provision of pain care in the UK

### The current landscape of pain services in the UK

In the UK, people living with persistent pain are typically supported by their General Practitioner (GP, physician), other health professionals (e.g., physiotherapists or pharmacists), or by multi-disciplinary pain clinics or pain management programmes (12). The service provision for people living with persistent pain is determined at a local level by the respective funding organisations and may be in community, primary or secondary care settings. Typically, people present to a GP or first contact physiotherapist who has authority to refer patients to secondary care for more specialist support, although there is an absence of secondary pain clinic provision in some areas. Core standards for pain management services in the UK published by the Faculty of Pain Medicine provide a comprehensive overview of commissioning, pathways, personnel, interventions, and governance (13).

Biopsychosocial approaches to pain management are considered optimal (14), although fragmentation of bio-psycho-social elements of service delivery are recognised limitations (15, 16), especially when they are not part of the same referral “pathway” with oversight and organisation from one provider. There is variability in chronic pain service provision in different areas of the UK and this may be associated with health inequalities and inequities resulting in poor outcomes for patients (17, 19). Integrating care requires resources, commitment across organisations, functioning information technology, coordination of finances and care pathways, aligned objectives, and buy-in from teams (20).

### Rationale for system change

Between one-third and one-half of the UK population (28 million adults) are affected by persistent pain (21), creating significant pressures on NHS clinical services. Waiting times for NHS pain services are often many months. There is a need to ease pressures on clinical services yet also provide high quality care. The NHS long term plan is to give people more control of their own health with a drive for more personalised care. Personalised care aims to fundamentally shift how professionals work alongside patients and to focus on “what matters” to individuals (1). Working collaboratively across the voluntary, community and clinical sector is one way to achieve this.

A Cochrane review provided evidence that personalised care resulted in health improvements for long term conditions, including pain (22). Personalised care improved musculoskeletal health related quality of life, understanding of the condition and confidence in self-management for people living with fibromyalgia (23). A key feature of personalised care is to work in partnership with patients, as a whole person within the context of their whole life, to deliver care and support that matters most to individuals (24). Importantly, patients should have access to various support options including peer support and community-based resources to build knowledge, skills and confidence to manage their health and wellbeing (24). Social prescribing is a key component of personalised care. Social prescribing connects people to activities, groups, and services in their community to meet the practical, social and emotional needs that affect their health and wellbeing (25). The focus is to support individuals to have more control and choice over their health.

### Evidence to support system change

In the UK, the National Institute for Health and Care Excellence (NICE) make various recommendations to assess and support people living with chronic (primary and secondary) pain, including options accessible via community-based organisations (26). Previously, integrating community support (i.e., the voluntary and community sector) into NHS pathways has been challenging. However, the long-term plan of the NHS is to build the infrastructure for increased community engagement. The NICE were unable to evaluate the clinical and cost effectiveness of social interventions for persistent pain because there were no clinical studies comparing social interventions with standard care (27). However, a systematised review of social prescribing in the UK as part of non-clinical community interventions identified 86 schemes, of which 40 schemes were evaluated using either quantitative, qualitative or mixed methods approaches (28). Findings suggested benefits for self-esteem, confidence, mood, anxiety, depression, and mental well-being. Furthermore, a qualitative study by Moffatt et al. (29) found that a link worker social prescribing programme to connect people with long-term conditions, including pain, to the community, reduces social isolation and improves self-confidence, resilience, effective problem-solving strategies and health-related behaviours. There are increasing calls for healthcare to connect people to activities, groups, and services in their community because “The Biomedical Model [for treating pain] Needs Urgent Help” (30) p. 263. There are examples of voluntary and community sector support services working in partnership with the NHS specifically for people living with pain [e.g., Dover Town Primary Care Network (31)], but, to our knowledge, there are no NHS commissioned pain services that are voluntary and community sector-led. Sim and Barker contend that community pain services can be delivered at lower costs while enabling long-lasting integration of self-management into the lives of individuals (32).

## The concept of the Rethinking Pain service

Rethinking Pain is a new community-based pain service that aligns to the key features of personalised care. The Rethinking Pain service aims to support people's physical, mental, social, and environmental needs in the community, whilst providing a seamless pathway of care into therapy-led provision for people who need additional support. The purpose is to provide a central, holistic, connected, and accessible pain pathway for adults who have been experiencing pain that has been adversely affecting their quality of life, despite treatment, for greater than three months.

The Rethinking Pain service has been commissioned by Bradford District and Craven Health and Care Partnership, including funding from Primary Care Networks. Commissioning the voluntary and community sector to lead and deliver pain care, in partnership with the clinical sector, is a significant system change for this geographical area of England, i.e., this is a voluntary and community sector-led (rather than therapy- or medically- led) pain service.

The Rethinking Pain team comprises health coaches, social prescribers and community partners who are trained to support the health and wellbeing of people with persistent pain. Their role is “patient facing” i.e., they work in the community (in non-clinical settings) in partnership with people who are experiencing persistent pain. The Rethinking Pain team also comprises GPs and clinical therapists who have specialist experience and/or training in supporting people with persistent pain. Their roles are to support the health coaches, social prescribers, and community partners; to develop resources and educational materials; to provide clinical governance; to discuss more complex patients in regular multidisciplinary team meetings; and to deliver Cognitive Behavioural Therapy (CBT) with patients who require additional support.

Health coaches and social prescribers are recruited from local recruitment and community sector platforms to meet professional standards and competencies set out in the NHS Workforce development framework for health and wellbeing coaches. Health coaches complete a comprehensive in-house pain focussed training programme as part of their role at Rethinking Pain. This includes training on supported self-management for people living with persistent pain designed and delivered by clinical members of Rethinking Pain, supplemented by external courses and resources for practitioners (e.g., 10 Footsteps to Live Well With Pain, a training course for practitioners wanting to develop key skills and tools to support pain self-management; https://livewellwithpain.co.uk/ten-footsteps-programme/).

The design, delivery and development of the Rethinking Pain programme was informed through proactive engagement with caregivers, families, and the wider community via a variety of community consultation exercises. Service users are encouraged to involve family members, caregivers, or friends by attending educational modules, or being present during consultations, so that those living with people experiencing pain also receive relevant information about persistent pain. Choice and consent are important, and an interpreter service is available for individuals who do not speak English as a first language. In terms of the wider community, the Rethinking Pain team have partners from the voluntary and community sector who run engagement events in community settings and faith centres, including the delivery of educational modules.

### Referral process

**Figure 1:**
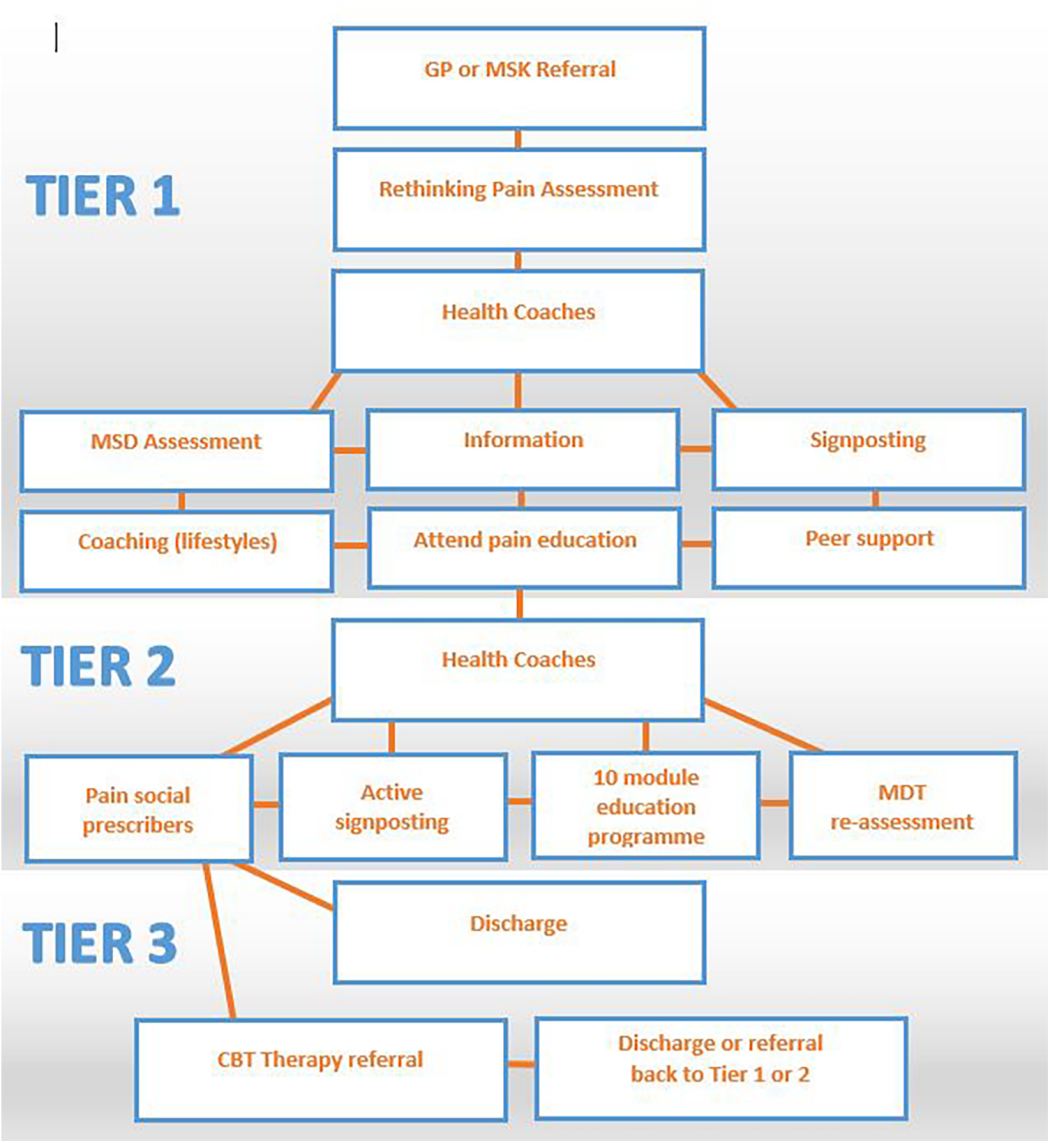
Rethinking pain referral pathway.

There is a central referral pathway for Primary Care Networks (PCNs) which enables GPs or musculoskeletal practitioners to refer people into the Rethinking Pain service. Once in the service, there are three tiers of support. An overview of the referral pathway is provided in Figure 1. In Tier 1, pain trained health coaches provide 1–1 support. They work with individuals to understand what is important to them in the context of their whole life. Individuals are signposted to a 2 h education module and are connected into the community. People who require additional support move through to Tier 2 where there is more active signposting from health coaches and/or social prescribers plus a range of education modules. Tier 2 education modules are continually being developed as the Rethinking Pain team consult different communities within Bradford District and Craven. Currently, the Tier 2 modules are:

1. More on Managing Pain
2. Keeping Active & Safe Movement
3. Sleep Therapy
4. Emotional Wellbeing Support
5. Developing Helpful Habits, Setting Goals & Making Plans
6. Diet Therapy
7. Creative Therapies
8. Your Stories (a chance to talk and be heard)
9. Acceptance & Taking Control of Your Pain
10. Beliefs, Spirituality, Faith, and Pain.

In Tier 1 and Tier 2 the GPs and clinical therapists work “in the background” to support the health coaches, social prescribers, and community providers, and provide clinical governance. There are regular multi-disciplinary team (MDT) meetings where any patients that are requiring additional support are identified so that they can be discussed and where appropriate move seamlessly through to Tier 3 (Figure 1). The additional support offered in Tier 3 is therapy-led CBT.

### The uniqueness of the Rethinking Pain service

The salient driving force in the Rethinking Pain service is the voluntary and community sector. This is a step-change of traditional models of care which is highly contingent on statutory providers. Of course working with clinical practitioners remains a fundamental component, but this partnership is on a more equal footing and guided by advocating for wider system change. The health coach is a critical role, given that they can build trust and rapport and give time and resource to working in collaboration with the patient in non-medical settings:

“*Those health coach contacts are the person who’s with them on this longer and sustained journey, without a focus on discharge*” (Rethinking Pain team)

The health coach can be responsive to need and to negotiate goals and targets to support pain management, underpinned by principles of personalised care:

“*It really is a negotiation with the individual…their plan will be constantly setting goals, resetting the goals and it really is that relationship which is the core of it all*.” (Rethinking Pain team)

The Rethinking Pain service places great value on being socially connected and reconfiguring notions of biomedical cure by focussing on social, community and environmental needs. Rethinking Pain opts for a discourse on new possibilities and empowering individuals:

“*Supporting and empowering individuals so that they can better manage their pain in their daily lives…. from an individual’s perspective it’s equipping them with tools and giving them the confidence to try something new around managing their pain.*” (Rethinking Pain team)

“*Pain is inevitable to some extent, but it is the suffering element that I think we're trying to help people who are in pain manage better…..also helping them [the patient] live better with pain.*” (Rethinking Pain team)

A unique feature of the Rethinking Pain service is the seamless partnership and care pathway across the voluntary, community and clinical sector. This is a significant system change for Bradford District and Craven and if successful has potential to be scaled-up nationally. Reconfiguration of mindset and funding is necessary to realise system-change and “true cultural transformation” for persistent pain across the health sector globally (4). In the UK, health campaigns targeting policy makers and the public are changing the way people think about, talk about, and treat persistent pain. Perhaps the most critical systemic barrier to implementing Rethinking Pain on a larger scale is funding, particularly for services that are additional to existing provision, and/or innovative, and/or are awaiting the outcome of formal service evaluation. Prior to Rethinking Pain a community-barrier was people having the confidence to access voluntary and community activities without support. Rethinking Pain overcomes this using health coaches, social prescribers and link workers who gain the trust of the service user and act as a bridge between clinic and community. Moreover, there needs to be an uplift in the status of the voluntary and community sector by placing them as key partners so that they are engaged on an equal footing to the clinical team, with investment in training so that the expertise of health coaches, social prescribers and link workers is valued equally to that of clinical colleagues.

Reconfiguration from medical- or therapy-led to voluntary and community sector-led pain service provision was driven in part by a desire to de-medicalise through community engagement, pain education, health coaching, social prescribing, *and* tiered support in a single pathway of care. The intention of the Rethinking Pain service is to optimise the delivery of “whole-health”:

“*Working with clinical teams, that’s unique and that’s about a focus to bring this service to people in their place, in their communities where they're comfortable and where we can deliver in ways that meet their needs. I think it’s innovative…there is a lot of interest in this piece of work because of the system change potential and the learning around connecting services, and what happens for those services and for people in the service.*” (Rethinking Pain team)

### Service aspirations

Prior to Rethinking Pain, Bradford District and Craven services had the following challenges:

1. GPs had limited options for people with persistent pain
2. The number of people with persistent pain was increasing
3. Social determinants of persistent pain were not being addressed
4. Health inequalities and persistent pain

Bradford District and Craven Health and Care Partnership are addressing these needs by commissioning the voluntary and community sector to deliver a central, holistic, connected, accessible care pathway. The principal tenet of the Rethinking Pain team's core set of values is that the biomedical model has significant limitations and that health services alone are unable to counteract persistent pain in communities:

“*The medications we (health professionals) use are pretty useless (for chronic pain) and some of the interventions that we've thought of being quite successful in the past, again not great, and so therefore we as a district had to think about what else is there available to our chronic pain patients. And the honest answer was there was nothing.*” (Rethinking Pain team)

“*The answer isn't solely medical. If it was, we wouldn't need this service*.” (Rethinking pain team)

The Rethinking Pain service harvests the power of the voluntary and community sector, whilst simultaneously collaborating across the clinical sector, so that a person's physical, emotional, social, and environmental needs are considered.

“*Pain is multifactorial, multifaceted and managing everything else around the pain, including mental health, physical health….if you're able to manage those conditions and those factors, then you're more likely to have a better quality of life….*” (Rethinking Pain team)

“*You know, the people sat around told us that they've tried all the medicines. They've done acupuncture, they've done all of that. We explore how can we [Rethinking Pain] help you manage that [alternatives to medication] moving forward?*” (Rethinking Pain team)

A core ambition for the Rethinking Pain team is to meet diverse needs by proactively engaging with local communities representing such diversity. The team recognise the impact of health inequalities and the importance of addressing these for people living with persistent pain.

*For the (RP) service, to try and evaluate whether we're having an impact on our disadvantaged communities and (to consider) how we could change and adapt to cater for different needs. The population in Bradford is very diverse. It would be really good to try to see where we are really with those communities, so we can improve the offer to them.* (Rethinking Pain team*)*

Genuine co-production of the Rethinking Pain service is seen as central to succeeding in understanding and addressing health inequalities:

“*Moving forward, it (co-production) would be a real opportunity, to really grasp that with both hands and really involve the people that are accessing and using the service in developing it*” (Rethinking Pain team)

### The importance of cultural-adaptation

Rethinking Pain is based on community and voluntary participation within a multi-cultural region of the UK Rethinking Pain serves an ethnically diverse city with a significant Black, Asian and minority ethnic population, and a predominance of people with Pakistani heritage and Muslim faith. Bradford is ranked the third highest for health inequality nationally and characterised as a population group amongst the most income and employment deprived. Social and cultural conditions influence the prevalence, presentation, illness behaviour, and community responses to persistent pain in diverse communities. It is common for people with persistent pain in Bradford to experience life with high levels of social deprivation, poor housing and limited formal education, and the Rethinking Pain Team recognise that participants with persistent pain conceptualise and experience pain in a much wider sense than a Westernised biopsychosocial model. Service users frequently referred to “faith and belief” constructs to understand and live with pain, effectively using their faith and beliefs to live a positive life with pain. Thus, the nature and cultural utility of the educational modules delivered by Rethinking Pain have evolved over time to acknowledge and explore beliefs related to pain, e.g., the development of an educational workshop on Beliefs, Spirituality, Faith and Pain. Health coaches record and flag people they work with where conversations indicate their faith would be a barrier to them from engagement with aspects of the support. Health coaches attend bi-weekly meetings with a multidisciplinary team (MDT) that includes voluntary and community sector leads, a clinical team (GPs and CBT therapists), and social prescribers to discuss their caseloads and clients presenting with complex circumstances. The MDT meetings are an opportunity for the full Rethinking Pain team to support health coaches in their decisions and to consider and discuss the diverse offer of the programme.

Other aspects considered critical to the successful delivery of the Rethinking Pain service include; recruiting staff who are multilingual and of similar faith and representative of Bradford's diverse communities, a key partnership with a voluntary and community sector organisation Happy Healthy You (Bradford and Beyond), promoting voluntary and community sector engagement events to connect and work with the various communities represented in the region, and co-production of materials, sessions and workstreams with members of the community, to assure cultural appropriateness and sensitivities. Rethinking Pain uses an organisation with expertise to make information accessible to communities with low level literacy skills or where English is not the primary language.

### Service evaluation

The Rethinking Pain service will be evaluated by an independent research team, underpinned by principles of theory of change (11) that will reveal the principles, values, components, structures, processes, and goals of Rethinking Pain. This might overcome some of the potential barriers to scalability by showing the value of working with the voluntary and community sector and which aspects of the service may be transferable to different contexts and geographical areas. Funding is more likely to follow if Rethinking Pain demonstrates success at easing pressure on GP and hospital services. In addition, the Rethinking Pain team have instigated ten community consultations to be thematically analysed as part of the development and evaluation of the first year.

Findings from the development, implementation and evaluation of the service will be shared with commissioners, clinical, voluntary and community partners, patients, and the wider society. Evaluation activities will include theory of change workshops, interviews with key stakeholders/staff and partners, quantitative and qualitative analysis of monitoring data, and consultation with different communities.

## Implications for future policy and practice

A review by Kozlowska et al. (20) recognised resistance to change and constrained resources as a barrier to better integrated care in the UK Barriers included lack of commitment to integration by the organisations involved, conflicting organisational interests, insufficient resources to develop the integrated service, and inadequate mechanisms of payments between the organisations. We advocate Gaudet et al.'s call for “true cultural transformation” of services to a whole health system, if “whole-person health” with or without pain is to be realised (4). In that regard, the Rethinking Pain service will be tested across twelve primary care networks. If successful, the Rethinking Pain service will provide evidence for a new conceptual model for a health and well-being pathway that complements and reduces the need for medical intervention, and this can be adopted across the NHS, and potentially other long term health conditions.

Rethinking Pain provides opportunities for greater shared understanding of how the voluntary and community sector can work collaboratively with medical professionals and clinical settings to support patients with persistent pain. It is expected that a set of principles for targeting equitable interventions towards those with the greatest need and who face health inequalities, will be produced, and shared with the potential to be transferable and scalable. Successful development and implementation of integration of the Rethinking Pain service across the NHS will require shared goals and values across organisations and well-resourced teams to foster commitment and enthusiasm for joint working (20).

## Conclusion

Our perspective is that greater use of the voluntary and community sector, in partnership with the clinical sector, creates the conditions for a “whole-person” approach to pain management, leading to greater personalised care, whilst having the potential to ease some of the pressures on General Practitioners and other clinical services. The Rethinking Pain service are piloting this perspective, by providing innovative voluntary and community sector-led system change for people living with persistent pain in Bradford District and Craven. The Rethinking Pain team are adept at using de-medicalised pain “language” in de-medicalised settings and embracing the whole context of a person's living experience within their community. The intention is that people reconceptualise pain beyond a biomedically-dominated narrative and are empowered to embark on a “healing journey” meaningful within their socio-cultural context and grounded in the principles of salutogenesis. Evaluation of the Rethinking Pain system change will be published in subsequent articles by our team.

## Data Availability

The original contributions presented in the study are included in the article/Supplementary Material, further inquiries can be directed to the corresponding author.
